# Evaluating the value of lactic dehydrogenase in anti-MDA5-positive Dermatomyositis associated with interstitial lung disease

**DOI:** 10.3389/fmed.2026.1767474

**Published:** 2026-02-20

**Authors:** Wenhan Huang, Feifeng Ren, Kechen Qian, Yanqiu Jiang, Yichen Zhang, Lin Tang

**Affiliations:** Department of Rheumatology and Immunology, The Second Affiliated Hospital of Chongqing Medical University, Chongqing, China

**Keywords:** anti-MDA5-positive DM, anti-Ro-52 antibodies, interstitial lung disease, lactic dehydrogenase, serum ferritin

## Abstract

**Objective:**

This retrospective cohort study aimed to investigate the relationship between lactic dehydrogenase (LDH) and anti-melanoma differentiation-associated gene 5 antibodies-positive dermatomyositis (anti-MDA5-positive DM) associated with interstitial lung disease (ILD).

**Method:**

A total of 168 anti-MDA5-positive DM patients with ILD underwent comprehensive evaluation, including LDH, anti-Ro-52 antibodies, serum ferritin determination, and lung high-resolution computed tomography (CT).

**Results:**

The levels of LDH were significantly elevated in anti-MDA5-positive DM patients compared with the control group (*p* < 0.01). Anti-Ro-52-positive patients exhibited significantly higher LDH levels than anti-Ro-52-negative patients (*p* < 0.01). Patients with elevated serum ferritin exhibited markedly increased LDH levels compared with those with lower serum ferritin (*p* < 0.01). After treatment, LDH levels significantly increased in patients with ILD deterioration (*p* < 0.01), whereas LDH levels decreased in patients with ILD amelioration or stabilization (*p* < 0.01). The LDH levels were significantly elevated in the death group compared to the survival group before treatment (*p* = 0.02).

**Conclusion:**

(1) The LDH levels were affected by anti-Ro-52 antibodies and serum ferritin. (2) The severity of ILD in anti-MDA5-positive DM patients was positively correlated with LDH, suggesting that LDH might serve as an effective assessment indicator to evaluate the changes in ILD in these patients. (3) Elevated LDH levels were a crucial indicator of poor prognosis in anti-MDA5-positive DM patients, warranting close attention from rheumatologists.

## 1 Introduction

Sato et al. (1, 2) initially characterized anti-melanoma differentiation-associated gene 5 antibodies-positive dermatomyositis (anti-MDA5-positive DM); this disease has garnered considerable clinical attention owing to its propensity for rapidly progressive interstitial lung disease (RP-ILD) development, culminating in elevated mortality rates (3, 4). Undoubtedly, pulmonary assessment in anti-MDA5-positive DM patients is the core of the clinical work. Contemporary evaluation modalities predominantly encompass chest CT and pulmonary function assessments. Furthermore, patients showing elevated serum Krebs Von den Lungen-6 levels exhibit heightened predisposition toward RP-ILD development (5). Nevertheless, an urgent clinical imperative persists for a straightforward, efficacious indicator facilitating ILD severity assessment in anti-MDA5-positive DM patients.

Lactic dehydrogenase represents a pivotal redox enzyme catalyzing pyruvate-to-lactate conversion. LDH is abundantly distributed in tissues of the heart, the liver, the skeletal muscle, and the lungs. When cells were damaged or ruptured, enhanced permeability facilitated LDH release into systemic circulation, thereby elevating serum concentrations. Consequently, LDH serves as a significant tissue injury biomarker. Currently, LDH detection finds widespread application in myocardial infarction (6), liver injury (7), and tumors (8). Recent investigations have validated LDH’s crucial role in pulmonary injury evaluation, with LDH levels closely correlating with acute respiratory distress syndrome prognosis (9). Nevertheless, LDH research regarding anti-MDA5-positive DM-associated interstitial lung disease remains limited. This study retrospectively analyzed 168 anti-MDA5-positive DM patients from the Department of Rheumatology and Immunology at the Second Affiliated Hospital of Chongqing Medical University and explored the relationship between LDH and ILD to provide assistance for clinical guidance.

## 2 Methods

From September 2015 to July 2025, 168 anti-MDA5-positive DM patients with ILD were hospitalized at the Second Affiliated Hospital of Chongqing Medical University. Patients with comorbidities such as cardiac disease, skeletal muscle and myocardial involvement, and malignancies were excluded from the study. Pulmonary infections were excluded using sputum culture, procalcitonin test, serum galactomannan detection, (1,3)-β-D-glucan detection assay, Epstein–Barr virus DNA test, and human cytomegalovirus DNA test. The control group comprised individuals who underwent health examinations at the same institution. In accordance with the Helsinki Declaration, surviving patients and relatives of deceased patients provided informed consent for participation and data publication. The Ethics Committee of the Second Affiliated Hospital of Chongqing Medical University approved this study.

LDH levels were quantified using the lactic acid substrate methodology (reference range: 120–250 U/L; Fosun Diagnostics, Shanghai, China). Anti-MDA5 and anti-Ro-52 antibodies were tested using OMRMUN assay kits (EUROIMMUN, Beijing, China). Serum ferritin levels were measured using the chemiluminescence method using an Access Ferritin kit (reference range: 11–306 ng/mL; Beckman Coulter, CA, USA).

Grouping: (1) LDH levels were evaluated by comparing 168 patients with anti-MDA5-positive DM to the control group before treatment. (2) High-resolution pulmonary computed tomography was performed in 168 patients with anti-MDA5-positive DM before and after treatment to assess the relationship between LDH and ILD. Based on radiological findings, patients were dichotomized: the improvement group (*n* = 116) showed reduction or no significant changes in pulmonary lesions after treatment and the worsening group (*n* = 52) exhibited newly emerged infiltrative shadows in the lungs on post-treatment imaging, defined as disease progression, excluding factors such as infection, pulmonary embolism, or heart failure (10). All CT evaluations were conducted by an experienced radiologist and respiratory specialist. (3) The 168 anti-MDA5-positive DM patients were stratified according to anti-Ro-52 antibody status, ferritin concentrations, and prognostic outcomes to examine LDH variations.

For stable patients, LDH levels and lung CT were reevaluated after receiving treatment for 3 months. For patients with exacerbations, the above examinations were rechecked after receiving treatment for 2 weeks or 1 month.

### 2.1 Statistical analysis

All analyses used SPSS 19.0 (IBM, Armonk, NY, USA). Normally distributed data were presented as mean ± standard deviation, while non-normal distribution data were expressed as median and interquartile range. The Mann–Whitney test was used to compare the two groups. The Wilcoxon test was used to compare the difference before and after treatment. Multiple comparisons were assessed using the Kruskal–Wallis test and Dunn’s post-hoc test. Values of P of < 0.05 were considered significant.

## 3 Results

### 3.1 Baseline information

**Figure 1:**
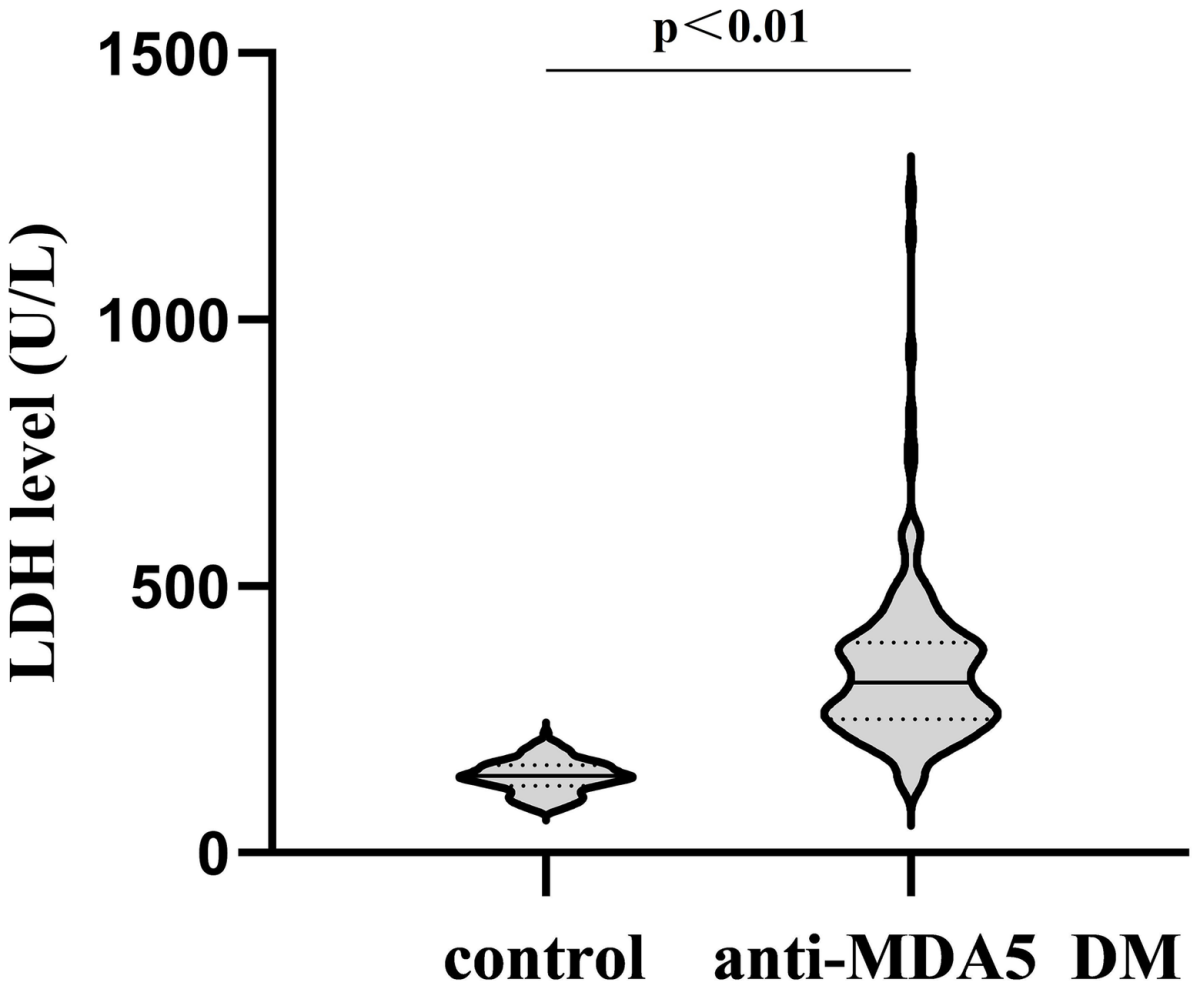
Comparison of LDH levels between the control group and patients with anti-MDA5-positive DM before treatment, *p* < 0.01.

The normal group encompassed 160 individuals (52 male participants, 108 female participants; male: female ratio: 1:2.07; mean age: 51.3 ± 9.6 years). The total number of anti-MDA5-positive DM patients was 168, including 57 male and 111 female patients (male: female: ratio 1:1.95; mean age: 51.7 ± 13.0 years) (Table 1). The LDH level was 143.5 ± 30.2 U/L in the control group and 319 U/L (250.2, 394) in patients with anti-MDA5-positive DM before treatment, which was significantly elevated versus controls (*p* < 0.01, Figure 1).

**Table 1:** Characteristics of 168 patients with anti-MDA5-positive DM and the control group.

	Control cohort (*n* = 160)	Anti-MDA5-positive DM cohort (*n* = 168)
Male	52	57
Female	108	111
Age	51.3 ± 9.6	51.7 ± 13.0
Steroid pulse	–	16 (9.5%)
Prednisolone	–	168 (100%)
IVIG	–	62 (36.9%)
Cyclophosphamide	–	101 (60.1%)
Mycophenolate mofetil	–	11 (6.5%)
Tacrolimus	–	83 (49.4%)
Cyclosporine	–	8 (4.8%)
JAK inhibitor	–	25 (14.9%)
Methotrexate	–	13 (7.7%)

Steroid pulse: methylprednisolone 500 mg daily for 3 days. IVIG, intravenous immunoglobulin.

### 3.2 The relationship between LDH and anti-Ro52 antibody

A total of 168 anti-MDA5-positive DM patients were dichotomized based on anti-Ro-52 antibody status. The results revealed: (1) among the 111 patients with anti-Ro-52 antibody, the LDH level before treatment was 336 U/L (267, 403). In contrast, among the 57 patients without anti-Ro-52 antibody, the LDH level before treatment was 299.6 ± 107.7 U/L. A statistically significant difference was observed between the two groups, *p* < 0.01. (2) The LDH level of patients with positive anti-Ro-52 antibodies was 279 U/L (193, 482) after treatment, which was not significantly different from that before treatment (*p* = 0.45). (3) The LDH level of patients without anti-Ro-52 antibodies was 203 U/L (156, 776) after treatment, which was significantly reduced compared to that before treatment (*p* < 0.01, Table 2).

**Table 2:** Comparison of LDH levels in patients with anti-MDA5-positive DM with or without anti-Ro-52 antibodies before and after treatment.

	Anti-Ro-52 antibodies positive cohort (*n* = 111)	Anti-Ro-52 antibodies negative cohort (*n* = 57)	*p* value
Before treatment (U/L)	336 [(267, 403)]	299.6 ± 107.7	<0.01
After treatment (U/L)	279 [(193, 482)]	203 [(156, 776)]	<0.01
*P* value	0.45	<0.01	

### 3.3 Relationship between LDH and serum ferritin

**Figure 2:**
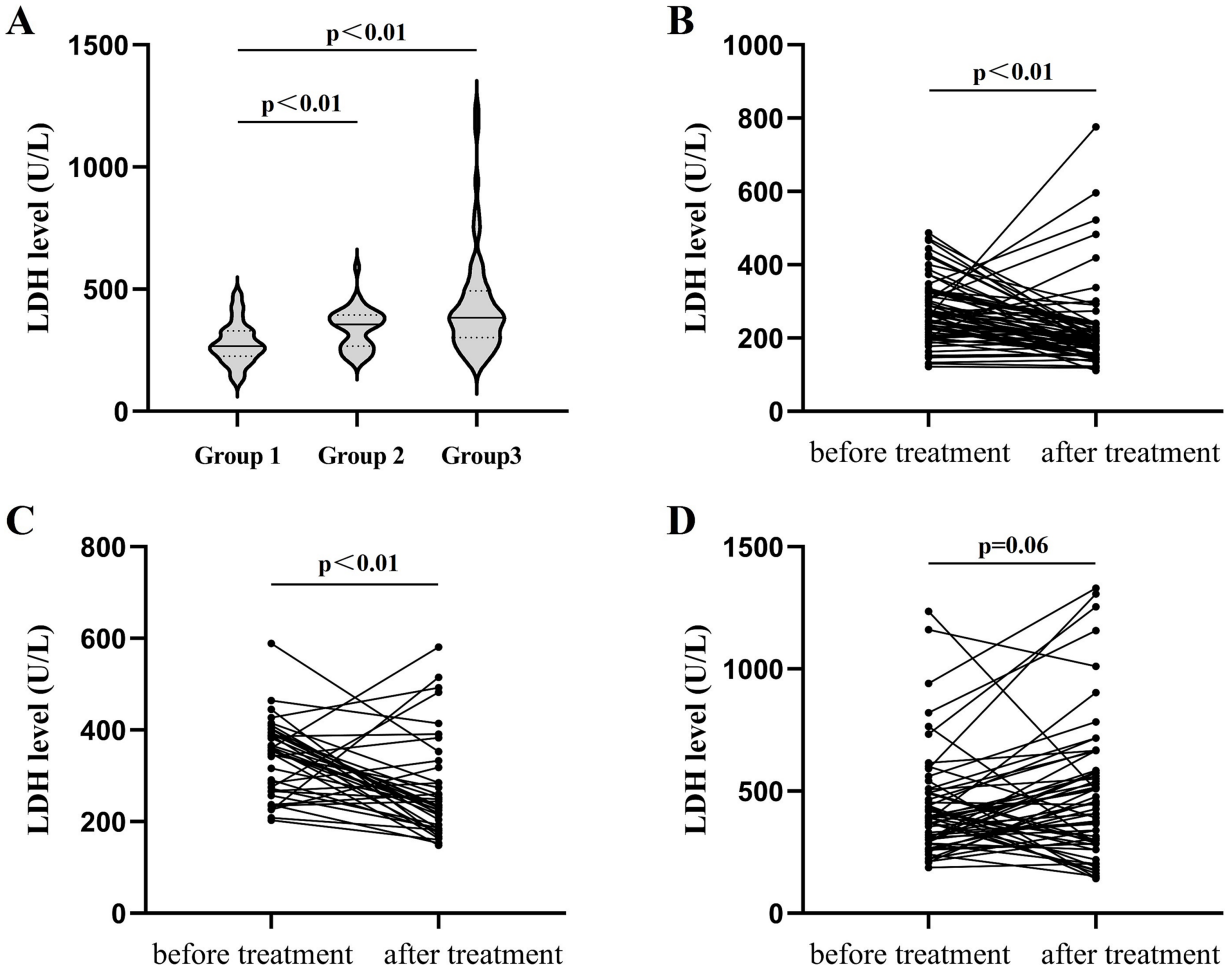
Relationship between LDH and serum ferritin. **(A)** Levels of LDH before treatment grouped by serum ferritin. **(B)** Changes in LDH levels before and after treatment for patients in Group 1 (serum ferritin < 500 ng/mL, *n* = 69). **(C)** Changes in LDH levels before and after treatment for patients in Group 2 (500 g/mL ≤ serum ferritin < 1,000 ng/mL, *n* = 39). **(D)** Changes in LDH levels before and after treatment for patients in Group 3 (1,000 g/mL ≤ serum ferritin ≤ 1,500 ng/mL, *n* = 60).

A total of 168 anti-MDA5-positive DM patients were categorized into three groups based on their ferritin levels. Group 1: serum ferritin<500 ng/mL (*n* = 69); Group 2: 500 ng/mL ≤ serum ferritin < 1,000 ng/mL (*n* = 39); and Group 3: 1,000 ng/mL ≤ serum ferritin≤1,500 ng/mL (*n* = 60). The LDH level of patients in Group 1 was 278.2 ± 84.6 U/L before treatment, significantly lower than that of patients in Groups 2 and 3 (Group 2: 340.4 ± 82.9 U/L; Group 3: 382.5 [301.3, 492.5 U/L]); respectively (*p* < 0.01; Figure 2A). After treatment, patients in Group 1 exhibited an LDH level of 193 U/L (158, 232), which was significantly lower than that before treatment (*p* < 0.01; Figure 2B). The LDH level of patients in Group 2 was 234 U/L (186, 318) after treatment, markedly diminished than that before treatment (*p* < 0.01; Figure 2C). The LDH level of patients in Group 3 after treatment was 435.5 U/L (285, 577), and the difference was not statistically significant than that before treatment (*p* = 0.06; Figure 2D).

### 3.4 Relationship between LDH and ILD

**Figure 3:**
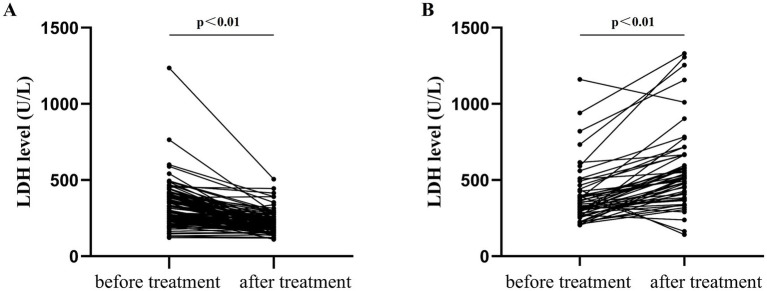
LDH fluctuations in 168 anti-MDA5-positive DM patients before and after treatment. **(A)** Improvement group: ILD was improved or stabilized after treatment (*n* = 115) and **(B)** Worsening group: ILD was aggravated after treatment (*n* = 53), *p* < 0.01.

To elucidate LDH-ILD correlations before and after treatment, we divided 168 anti-MDA5-positive DM patients into two groups: the improvement group, encompassing patients with improved or stabilized interstitial lung disease after treatment (*n* = 115), and the worsening group, comprising patients with deteriorated interstitial lung disease after treatment (*n* = 53). In the improvement group, the LDH level of patients significantly decreased after treatment (before treatment: 297 U/L [242, 392] vs. after treatment: 201 U/L [174, 244], *p* < 0.01, Figure 3A). Conversely, the worsening group exhibited a significant increase in LDH relative to the baseline when interstitial lung disease deteriorated (before treatment: 342 U/L [279.5, 431.5] vs. after treatment: 512 U/L [395, 664], *p* < 0.01, Figure 3B).

### 3.5 Relationship between LDH and prognosis

A total of 168 anti-MDA5-positive DM patients were categorized into a survival group (*n* = 126) and a deceased group (*n* = 42) based on their prognosis. (1) The LDH level of patients in the survival group was 299.5 U/L (241.5, 392.3) before treatment, which was significantly lower than that in the deceased group (345.0 U/L [298.8, 472.0], *p* = 0.02). (2) After treatment, the LDH level in the survival group decreased to 203.5 U/L (174.8, 259.3), which was significantly lower than that before treatment (*p* < 0.01). (3) In the deceased group, the LDH level increased to 534.5 U/L (444.3, 666.5) after treatment, which was significantly higher than that before treatment (*p* < 0.01) (Table 3).

**Table 3:** Comparison of LDH levels before and after treatment in patients with anti-MDA5-positive DM based on prognosis.

	Survival patients of anti-MDA5-positive DM (*n* = 126)	Deceased patients of anti-MDA5-positive DM (*n* = 42)	*p* value
Before treatment (U/L)	299.5 [(241.5, 392.3)]	345.0 [(298.8, 472.0)]	0.02
After treatment (U/L)	203.5 [(174.8, 259.3)]	534.5 [(444.3, 666.5)]	<0.01
*P* value	<0.01	<0.01	

## 4 Discussion

Anti-MDA5-positive DM patients developing RP-ILD frequently exhibit poor prognosis and elevated mortality rates. However, lung lesion assessments in anti-MDA5-positive DM remain relatively scarce with inherent constraints, such as radiation exposure from repeated chest CT scans over a short period of time, the inability of critically ill patients to adequately complete pulmonary function assessments, and the limited practicality of KL-6 testing owing to economic constraints. Consequently, identifying efficacious biomarkers for evaluating ILD severity in anti-MDA5-positive DM patients assumes paramount significance.

Our findings revealed that the LDH levels in anti-MDA5-positive DM patients with ILD were markedly elevated compared with those in the control group before treatment, indicating that LDH is an important indicator that should not be ignored. Subsequently, we explored LDH correlations with anti-MDA5-positive DM comprehensively.

First, extensive research has validated the pivotal role of anti-Ro-52 antibodies in inflammatory myopathies. Patients with positive anti-Ro-52 antibodies were more likely to develop rapidly progressive interstitial lung disease (11), subcutaneous and mediastinal emphysema (12), and pharyngeal lesions (13). Our study revealed that the LDH level before treatment was significantly more elevated in patients with anti-Ro-52 antibodies than in those without anti-Ro-52 antibodies, confirming LDH-anti-Ro-52 antibody associations in anti-MDA5-positive DM patients. Furthermore, no significant differences were found in the LDH level of patients positive for anti-Ro-52 antibodies before and after treatment, whereas the level of LDH in patients negative for anti-Ro-52 antibodies decreased significantly after treatment. This is potentially attributable to more severe interstitial lung disease in anti-Ro-52-positive patients.

Second, serum ferritin is also an important evaluation indicator for anti-MDA5-positive DM. Takahisa et al. (14) suggested that anti-MDA5-positive DM represents macrophage activation occurring in the lungs, distinguished by pronounced ferritin elevation. By stratifying ferritin levels, we determined that LDH levels in anti-MDA5-positive DM patients exhibited concordance with ferritin levels, exhibiting a direct correlation between inflammatory magnitude and LDH elevation. Additionally, we found that, under different inflammatory states, the difference in LDH levels of anti-MDA5-positive DM patients was also observed before and after treatment. Patients with serum ferritin <500 ng/mL and 500–1,000 ng/mL showed significant post-treatment LDH attenuation versus baseline, signifying a better treatment response in mild-to-moderate inflammatory states. Conversely, when serum ferritin was between 1,000 and 1,500 ng/mL, no significant difference was found in LDH levels before and after treatment, indicating that glucocorticoids and immunosuppressants have no improvement effect on LDH in severe inflammatory conditions. This finding bears profound clinical implications, underscoring the imperative for rheumatologists to judiciously modulate inflammatory burden in anti-MDA5-positive DM patients, thereby attenuating disease activity and improving the prognosis.

Given anti-MDA5-positive DM’s predominant lung manifestations, we prioritized LDH-ILD correlational analysis. Our data indicated that LDH levels diminish relative to the baseline when ILD lesions are improved or stabilized post-treatment. Conversely, progressive ILD deterioration and intensified pulmonary injury precipitate substantial tissue LDH efflux into systemic circulation, markedly elevating serum concentrations versus pretreatment values. These observations establish a positive correlation between LDH levels and ILD severity in anti-MDA5-positive DM patients. On the one hand, extracellular LDH functions as a damage-associated molecular pattern, promoting the release of inflammatory factors and exacerbating pulmonary inflammation. On the other hand, LDH’s fundamental physiological role is to catalyze pyruvic acid into lactic acid. A high concentration of lactic acid in tissues can activate TGF-*β*, inducing myofibroblast differentiation and thus accelerating the pulmonary fibrosis process (15). Our study confirmed that LDH was a simple yet effective biomarker to evaluate the changes in ILD in patients with anti-MDA5-positive DM.

Finally, we found that the LDH levels were substantially more elevated in the deceased group than in the survival group before treatment. After treatment, LDH levels in the deceased group escalated further while exhibiting marked amelioration in the survival group, indicating LDH’s prognostic significance in anti-MDA5-positive DM. Analogous findings (16, 17) in COVID-19 patients characterized by acute lung injury showed that non-survivors had significantly higher levels of LDH than survivors. As a result, LDH was regarded as an important indicator for evaluating patients’ respiratory function and predicting their prognosis. Combined with the above findings and our study, it was strongly confirmed that LDH plays a pivotal role in lung lesion assessment in anti-MDA5-positive DM patients.

It is worth noting that this is a retrospective study; therefore, inherent limitations should not be ignored. First, our study exclusively examined LDH’s relationship with anti-MDA5-positive DM, precluding characterization of other inflammatory myopathy types, such as antisynthetase syndrome. Second, mechanistic exploration remains superficial, requiring deeper investigation. Finally, single-center clinical data necessitate multicenter validation with expanded sample sizes.

In conclusion, this study elucidated LDH’s associations with serological markers, lung interstitial lesions, and prognosis in anti-MDA5-positive DM patients, furnishing clinically relevant insights and establishing groundwork for subsequent research.

## Data Availability

The raw data supporting the conclusions of this article will be made available by the authors, without undue reservation.

## References

[1] Sato S Hirakata M Kuwana M Suwa A Inada S Mimori T . Autoantibodies to a 140-kd polypeptide, CADM-140, in Japanese patients with clinically amyopathic dermatomyositis. Arthritis Rheum. (2005) 52:1571–6. doi: 10.1002/art.21023, 15880816

[2] Sato S Hoshino K Satoh T Fujita T Kawakami Y Kuwana M. RNA helicase encoded by melanoma differentiation-associated gene 5 is a major autoantigen in patients with clinically amyopathic dermatomyositis: association with rapidly progressive interstitial lung disease. Arthritis Rheum. (2009) 60:2193–200. doi: 10.1002/art.24621, 19565506

[3] Yang Y Li Y Yuan W Zhang S He X Ji J. Risk factors for mortality in anti-MDA5 antibody-positive dermatomyositis with interstitial lung disease: a systematic review and meta-analysis. Front Immunol. (2025) 16:1628748. doi: 10.3389/fimmu.2025.1628748, 40746563 PMC12310698

[4] Li H Zou R Xin H He P Xi B Tian Y . Mortality risk prediction in patients with Antimelanoma differentiation-associated, gene 5 antibody-positive, Dermatomyositis-associated interstitial lung disease: algorithm development and validation. J Med Internet Res. (2025) 27:e62836. doi: 10.2196/62836, 39908093 PMC11840371

[5] Ye Y Fu Q Wang R Guo Q Bao C. Serum KL-6 level is a prognostic marker in patients with anti-MDA5 antibody-positive dermatomyositis associated with interstitial lung disease. J Clin Lab Anal. (2019) 33:e22978. doi: 10.1002/jcla.22978, 31301087 PMC6805307

[6] Wroblewski F Ruegsegger P Ladue JS. Serum lactic dehydrogenase activity in acute transmural myocardial infarction. Science. (1956) 123:1122–3. doi: 10.1126/science.123.3208.1122, 13324162

[7] Cassidy WM Reynolds TB. Serum lactic dehydrogenase in the differential diagnosis of acute hepatocellular injury. J Clin Gastroenterol. (1994) 19:118–21. doi: 10.1097/00004836-199409000-00008, 7963356

[8] Gallo M Sapio L Spina A Naviglio D Calogero A Naviglio S. Lactic dehydrogenase and cancer: an overview. Front Biosci. (2015) 20:1234–49. doi: 10.2741/4368, 25961554

[9] Zhang F Zhang M Niu Z Sun L Kang X Qu Y. Prognostic value of lactic dehydrogenase-to-albumin ratio in critically ill patients with acute respiratory distress syndrome: a retrospective cohort study. J Thorac Dis. (2024) 16:81–90. doi: 10.21037/jtd-23-1238, 38410562 PMC10894402

[10] Raghu G Collard HR Egan JJ Martinez FJ Behr J Brown KK . ATS/ERS/JRS/ALAT committee on idiopathic pulmonary fibrosis. An official ATS/ERS/JRS/ALAT statement: idiopathic pulmonary fibrosis: evidence-based guidelines for diagnosis and management. Am J Respir Crit Care Med. (2011) 183:788–824. doi: 10.1164/rccm.2009-040GL 21471066 PMC5450933

[11] Chen Y Chen YF Chiu LC Yu KH Chan TM. Coexistence of anti-melanoma differentiation-associated protein 5 and anti-Ro52 antibodies in patients with idiopathic inflammatory myopathy: a retrospective cohort study. Clin Rheumatol. (2025) 44:3605–13. doi: 10.1007/s10067-025-07562-1. Epub ahead of print, 40663261

[12] Huang W Chen D Ren F Luo L Zhou J Huang D . The clinical characteristics of subcutaneous and mediastinal emphysema in anti-melanoma differentiation-associated 5 positive dermatomyositis associated with interstitial lung disease. Clin Exp Rheumatol. (2024) 42:262–8. doi: 10.55563/clinexprheumatol/84kd56, 38147317

[13] Huang W Ren F Deng D Luo L Zhou J Huang D . The clinical characteristics of pharyngeal and laryngeal lesions in anti-MDA5-positive dermatomyositis patients. Clin Exp Rheumatol. (2025) 43:276–81. doi: 10.55563/clinexprheumatol/t0478a, 39625817

[14] Gono T Sato S Kawaguchi Y Kuwana M Hanaoka M Katsumata Y . Anti-MDA5 antibody, ferritin and IL-18 are useful for the evaluation of response to treatment in interstitial lung disease with anti-MDA5 antibody-positive dermatomyositis. Rheumatology (Oxford). (2012) 51:1563–70. doi: 10.1093/rheumatology/kes102, 22589330

[15] Kottmann RM Kulkarni AA Smolnycki KA Lyda E Dahanayake T Salibi R . Lactic acid is elevated in idiopathic pulmonary fibrosis and induces myofibroblast differentiation via pH-dependent activation of transforming growth factor-β. Am J Respir Crit Care Med. (2012) 186:740–51. doi: 10.1164/rccm.201201-0084OC, 22923663 PMC3480515

[16] Li G Xu F Yin X Wu N Li Y Zhang T . Lactic dehydrogenase-lymphocyte ratio for predicting prognosis of severe COVID-19. Medicine. (2021) 100:e24441. doi: 10.1097/MD.0000000000024441, 33530248 PMC7960489

[17] Gupta GS. The lactate and the lactate dehydrogenase in inflammatory diseases and major risk factors in COVID-19 patients. Inflammation. (2022) 45:2091–123. doi: 10.1007/s10753-022-01680-7, 35588340 PMC9117991

